# In Vitro Strategy for the Enhancement of the Production of Bioactive Polyphenols in Transformed Roots of *Salvia bulleyana*

**DOI:** 10.3390/ijms23147771

**Published:** 2022-07-14

**Authors:** Marta Krzemińska, Aleksandra Owczarek, Monika A. Olszewska, Izabela Grzegorczyk-Karolak

**Affiliations:** 1 Department of Biology and Pharmaceutical Botany, Medical University of Lodz, Muszynskiego 1, 90-151 Lodz, Poland; marta.wojciechowska2@stud.umed.lodz.pl; 2 Department of Pharmacognosy, Medical University of Lodz, Muszynskiego 1, 90-151 Lodz, Poland; aleksandra.owczarek@umed.lodz.pl (A.O.); monika.olszewska@umed.lodz.pl (M.A.O.)

**Keywords:** basal medium selection, genetic transformation, light spectra, plant biotechnology, rosmarinic acid, salvianolic acid K, TOPSIS method

## Abstract

The underground parts of *Salvia bulleyana*, a rare Chinese plant species, have long been used in traditional Chinese medicine. The *Rhizobium rhizogenes*-transformed root culture obtained from this plant might be a promising novel source of valuable phenolics, including rosmarinic acid. The present study identifies for the first time, the optimal growth conditions of *S. bulleyana* hairy roots regarding production efficiency. The comprehensive optimization comprised cultivation in different basal media (B5, SH, MS, and WP) with full- and half-strength macro- and microelements, different vitamin contents (full, half, one-quarter part, and without) and sucrose concentrations (2, 3, 4, 5%), and under different light conditions: in dark, under blue LED (λ = 430 nm), red LED (λ = 670 nm), mixed blue and red LED (30%:70%), and white LED (390–670 nm). Hairy root growth and bioactive compound accumulation were also detailed every five days over the 50-day culture cycle. The optimal conditions were determined using a technique for order preference by similarity to the ideal solution (TOPSIS). The most efficient combination for root growth and polyphenol content was found to be ½SH liquid medium with half vitamin concentration and 3% sucrose when grown in the dark. The biomass yield during the growth cycle was 6.1 g (fresh weight—FW) and 0.92 g (dry weight—DW) on one Erlenmeyer flask: a 14.3-fold increase in FW and 16.1-fold increase in DW in relation to the inoculum. The highest mean total phenolic content was 93.6 mg/g DW including about 70 mg/g DW rosmarinic acid, reached on day 40 of culture; compared to roots of two-year-old plants grown under field conditions, the total phenolic acid content was four times higher and rosmarinic acid eight times higher. The obtained results place the investigated culture among the best hair root cultures for rosmarinic acid production.

## 1. Introduction

*Salvia bulleyana* Diels, commonly named *Zi Danshen*, is one of the endemic Chinese *Salvia* species that overgrows the northwest hillsides of Yunnan Province [1]. Its roots have been used as an equivalent of Danshen (dried and micronized roots of *Salvia miltiorrhiza*) [2]. In traditional Chinese medicine, the phrase *Zi Danshen* means “to have healing properties”, and the plant is used locally to soothe irritability and insomnia [2]. It is also known as an effective herbal agent in coronary heart disease, and liver and kidney dysfunctions [2]. Two main classes of secondary metabolites have been isolated from the roots of *S. bulleyana*: phenols and tanshinones [2,3]. The main compounds of the raw material are two polyphenolic acids: rosmarinic acid (RA) and salvianolic acid K (SAK) [2,4,5,6].

Many studies have confirmed that RA has strong antioxidant properties [7]. Due to the current consumer concerns about the safety of synthetic preservatives, plant extracts rich in polyphenols such as RA are increasingly being used to extend the shelf life of food and cosmetics [8]. RA has already been investigated (with positive results) as an antioxidant in many food models, such as beverages [9], dairy products [10], processed meat [11], or edible oils [12]. Moreover, it has been found to demonstrate bacteriostatic and bactericidal activities, making it an even more promising candidate to be a natural preservative [13,14]. An additional advantage of RA is its health-promoting properties confirmed in many in vitro and in vivo models. The antiradical potential of RA together with antiinflammatory activity by inhibiting various enzymes, including lipoxygenase, cyclooxygenase, nitric oxide synthase, or myeloperoxidase, play a key role in its protective cellular effects [15,16]. The compound was found to decrease inflammatory cytokine expression by modulating key adipogenic transcription factors [17]. RA can also prevent and treat atopic dermatitis and allergies [18] and has demonstrated therapeutic potential against some cancers, e.g., colon, skin, or breast cancer [18] by a multidirectional mechanism, i.e., by inhibiting proliferation and migration and inducing apoptosis [19].

Due to these properties, the demand for RA and its industrial importance is growing. However, its content in the roots of various sage species rarely exceeds 1% of dry weight and is often many times lower [20]. Additionally, environmental fluctuations strongly affect plant growth and the phytochemical profile, resulting in qualitative and quantitative variations in secondary metabolites [21]. Meanwhile, plant biotechnology can offer alternative methods for obtaining valuable plant material with a more stable constituent profile and without degrading the environment. In vitro cultures are independent of geographical factors and seasons, allow for the elimination of biological contaminants (bacteria, fungi, viruses, parasitic insects), and offer efficient production in a shorter period of time than in traditional field conditions [22]. Moreover, in vitro cultures offer the possibility to increase the biosynthesis of bioactive compounds by simple strategies such as selecting highly-productive lines, modifying a medium’s components, or changing the physical conditions of the culture (temperature, lighting) [23].

One of the most studied and promising in vitro plant systems are hairy roots. They are characterized by having a high stability, and selected clones may demonstrate rapid growth in a medium without regulators [24]. In our previous research, we established a procedure for obtaining hairy root cultures of *S. bulleyana* that are rich in phenolic acids including RA [25] and might be developed into a novel promising source of those compounds. For this to happen, further improvement in the culture’s productivity should be performed by optimization of its growth conditions.

As a multicriteria decision making process, optimization of a culture’s performance is a complex task. Preferably, the optimal culture should be characterized by superior growth parameters and maximum production of all the important phytochemicals. However, it might not always be possible to maximize all the criteria simultaneously, as conditions promoting growth might not be ideal for metabolite production and vice versa. To facilitate the decision making in such cases, many statistical/mathematical tools have been developed. Attempts have also been made to use artificial intelligence models and optimization algorithms in order to improve plant tissue culture [26]. The methods are becoming more promising for modeling and optimizing complex systems to achieve better results in less time; however, despite their potential, their use is still limited due to complex definition terms and computational algorithms. Such complexity is not always required, and, if possible, simpler tools that allow satisfactory results to be obtained should be applied. A very simple tool that can be used for the selection of the best option from several available variants based on a couple of chosen parameters is the technique for order of preference by similarity to ideal solution (TOPSIS). It allows a solution to be selected from the set of alternatives that is closest to the theoretical ideal best option and farthest from the theoretical worst option. Among the advantages of the method are its objectivity, rationality, and simple computation process [27]. The method has been successfully applied in many areas of industry [27]; however, its potential in plant biotechnology has so far been underestimated.

The aim of this study was to optimize for the first time the growth conditions of the previously selected clone of *S. bulleyana* hairy roots as a novel source of phenolics. The study examines the influence of different media compositions and concentrations, and vitamin and sucrose contents, as well as the effects of various light conditions on secondary metabolite accumulation and the growth of the transformed root culture. In ambiguous cases, TOPSIS analysis was applied to identify the optimal conditions, which is a newly emerging approach in biotechnological studies. Finally, the research included a detailed analysis of culture growth and the production of bioactive compounds every five days over a 50-day growth cycle to determine the optimal harvest time.

## 2. Results

### 2.1. The Effect of Basal Medium on Culture Growth and Polyphenolic Acid Accumulation

**Figure 1:**
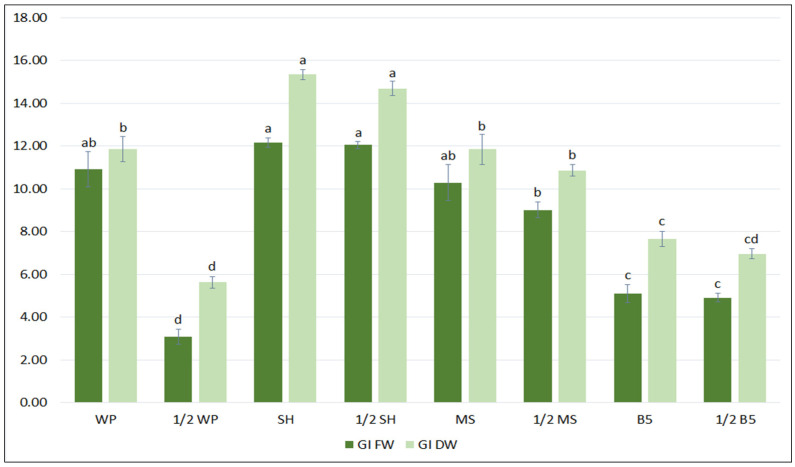
Growth indexes of transformed roots of *S. bulleyana* received for fresh (GI FW) and dry (GI DW) weight during cultivation in different media for five weeks. The results are mean values ± SE. Different letters indicate statistical differences between samples separately for FW and DW.

In the first step of the optimization process, the biomass accumulation, the qualitative and quantitative polyphenolic profiles of the *S. bulleyana* hairy root culture were determined following cultivation in different growth media for five weeks. Four different media (MS, WP, B5, SH), each in two concentration levels (full-strength, half-strength) were tested. As shown in Figure 1, hairy root growth was strongly affected by the media type. The maximum biomass increase was obtained when the roots were cultivated in full- or half-strength SH medium, with a GI of 12–12.2 for FW and 14.7–15.3 for DW. The roots demonstrated a more than 13-fold increase in FW and 16-fold in DW relative to an inoculum under optimal conditions, while in the least favorable condition the increase was 4-fold and 6.5-fold, respectively. Interestingly, apart from the basal WP media, no statistically significant differences in biomass accumulation were observed between the full and reduced macro- and micronutrient content for different medium types (Figure 1).

**Figure 2:**
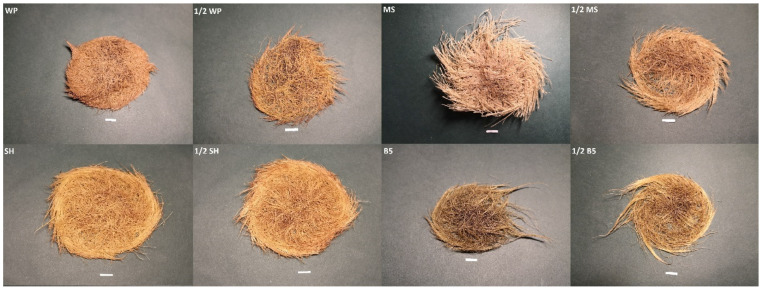
Transformed roots of *S. bulleyana* C4 clone cultivated in different media after five weeks (scale 1 cm).

Root morphology also differed according to medium type (Figure 2). The roots grown in the basal SH media were light brown and branched strongly with thin lateral roots; those grown in the WP media also branched strongly and had thinner roots but with shorter branches compared to the SH media. The roots grown in the basal MS media were the thickest with short branches and light beige in color. The roots from the basal B5 media demonstrated intermediate branch thickness and length, and were brownish grey in color; however, those from the full macro- and microsalts medium were darker than those in the half-strength medium.

The type of culture medium did not change the qualitative composition, with the same nine phenolic acids being identified in each analyzed material. Moreover, the predominant polyphenol in all samples was RA, which constituted 80–90% of all analyzed polyphenolic acids. However, the culture media had a significant impact on the content of particular compounds and total polyphenol accumulation (Table 1).

Our results show that SH medium-grown roots had the highest secondary metabolite levels: 79.2 mg/g DW total polyphenols, including 62.2 mg/g DW RA and 8.1 mg/g DW salvianolic acid K (SAK). The contents of the remaining seven phenolic acids were in the range of 0.4–2.3 mg/g DW. The TPC content of the roots cultivated in the SH medium was more than two times higher than in the MS medium, which turned out to be the least favorable in terms of bioactive compound accumulation. Both B5 media variants were strongly stimulating for RA biosynthesis (55.01–59.23 mg/g); however, they limited the biosynthesis of other analyzed compounds, especially SAK, whose content was eight times lower in roots cultivated in the B5 media compared to the SH medium (Table 1).

### 2.2. The Effect of Vitamin Content on Culture Growth and Polyphenolic Acid Accumulation

**Figure 3:**
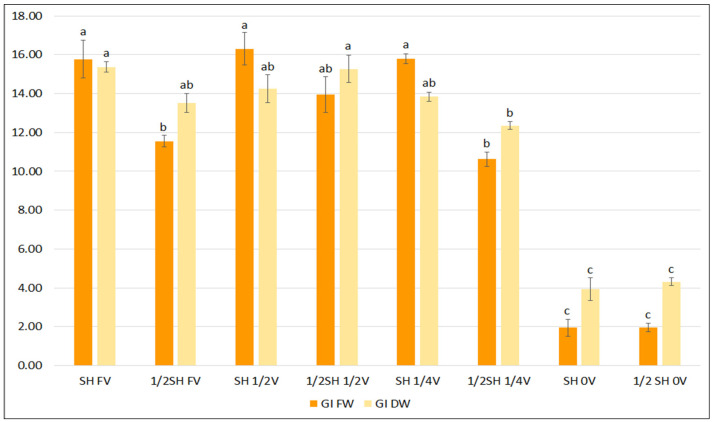
Growth indexes of transformed roots of *S. bulleyana* received for fresh (GI FW) and dry (GI DW) weight during cultivation in SH and ½SH medium supplemented with different concentrations of vitamins (FV—full vitamin, ½FV—half strength of vitamins, ¼FV—quarter of strength of vitamins, 0V—without vitamins) for five weeks. The results are mean values ± SE. Different letters indicate statistical differences between samples separately for FW and DW.

To determine the effect of vitamins in the basal medium, two media known to effectively enhance growth and phenolic acid production were selected: SH and ½SH. Lowering the concentration of vitamins and inositol in the media to one-half and one-quarter of the content did not inhibit the growth of *S. bulleyana* hairy roots, and even stimulated it in the case of ½SH with ½V (Figure 3). Only a complete lack of vitamins in the medium resulted in a statistically significant reduction in the FW and DW indexes for both studied media.

Lowering the level of vitamins in the SH medium resulted in a gradual decrease in the polyphenolic levels, both in the total contents and the levels of the three primary compounds: RA, SAE, and SAK. On the other hand, reducing the vitamin content in the ½SH medium to half, increased the TPC in the root culture by 30%, including MR by 55%, RAH by 44%, RA by 30%, SAK by 20%, and SAE by 170% compared to that grown in the ½SH medium with full vitamin concentration (Table 2). A further reduction in the vitamin concentration in the ½SH medium to one-quarter resulted in a decrease in the polyphenolic acid content, although their total content in the roots was still 17% higher than for the ½SH medium with full vitamins. As in the case of biomass accumulation, the complete removal of vitamins from the culture medium caused a drastic decrease in the TPC; however, the SAK content in those conditions was actually the highest (Table 2).

As the optimal medium could not be identified based on the productivity and growth parameters of the culture alone, the TOPSIS analysis was introduced (Table 3). Four parameters were selected as important to follow, including the DW growth index, TPC, RA content, and SAK content, the latter being the main representative of the salvianolic acids. To avoid unnecessary complexity, all of the parameters were considered equally important, and thus the weight of each was set to 0.25.

The highest performance score was found for the ½SH medium with ½ vitamin content, and hence this might be regarded as the optimal medium (Table 3). Indeed, the culture obtained on that medium demonstrated the highest TPC, with a DW growth index and RA content that were not significantly different to the highest variant, and with a reasonably high SAK content (6.61 mg/g) when compared to the best (7.84 mg/g) and the worst variants (1.52 mg/g). An additional advantage is also the lower cost of the medium in comparison to the full-strength option. Therefore, the ½SH medium with ½ vitamins was selected for further experiments.

### 2.3. The Effect of Sucrose Content on Culture Growth and Polyphenolic Acid Accumulation

**Figure 4:**
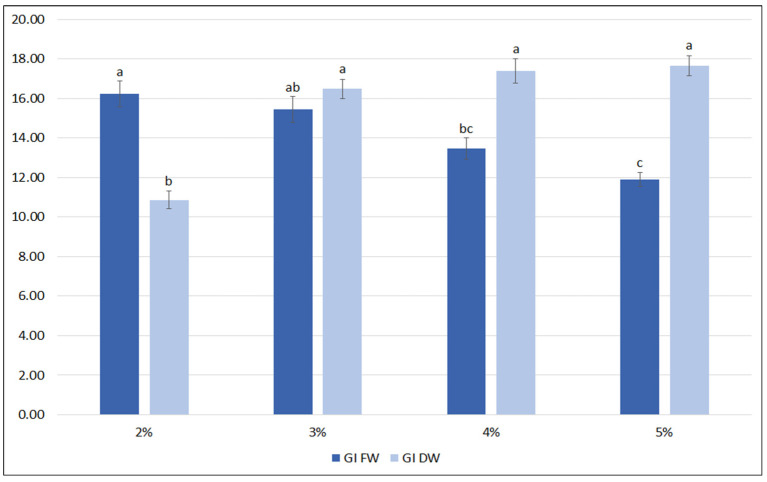
Growth indexes of transformed roots of *S. bulleyana* received for fresh (GI FW) and dry (GI DW) weight during cultivation in ½SH medium with ½ vitamin concentration supplemented with different sucrose concentrations (2–5%) for five weeks. The results are mean values ± SE. Different letters indicate statistical differences between samples separately for FW and DW.

A further optimization of the nutrient medium included the influence of sucrose level (2%, 3%, 4%, 5%). It was observed that the highest FW biomass was achieved when the hairy roots were grown in the medium with 2% and 3% sucrose (Figure 4). At higher sucrose concentrations, the FW of the roots significantly decreased. In contrast, the lowest DW was observed for roots cultivated in 2% sucrose, and this value increased by almost 50% for 3% sucrose, with no significant increase in DW being observed for the higher sucrose concentrations (4 and 5%); finally, about 1 g DW per flask was reached, with a growth index between 16.5 and 17.7. 

The highest polyphenolic acid production was recorded in hairy roots grown in the medium with 2% sugar supplementation (Table 4). A slight decrease was recorded following an increase in sucrose to 3%, but a further increase in the sucrose concentration caused a significant reduction in the TPC. This decrease was seen for most of the compounds, including RA, which demonstrated a 30% decrease for 4% and 5% sucrose compared to 2% sucrose. On the other hand, the level of some compounds such as SAK or RAH increased with sugar concentration, while MR remained at a similar level. The highest SAK content was reported in hairy roots in the presence of 5% sucrose (12.53 mg/g DW) (Table 4). This value was almost 2-fold higher than for 2% sucrose, and 30% higher than for 3% sucrose. The 4% sucrose content was optimal for RAH accumulation in the root culture, which peaked at seven times higher than observed for 2% sucrose, and 50% higher than for the 3% sugar medium.

As in the case of vitamin concentration, none of the sucrose levels yielded the best results for all productivity/growth parameters; therefore, the TOPSIS analysis was run again. The results indicate that the medium with 3% sucrose concentration demonstrated the highest overall performance (Table 5). In that case, the good DW accumulation managed to balance out and even supersede the slightly lower phenolic production achieved for that medium. This seems reasonable since a higher biomass might yield higher quantities of polyphenols. Therefore, the 3% sucrose variant was used for further experiments.

### 2.4. The Effect of Light Conditions on Culture Growth and Polyphenolic Acid Accumulation

The influence of different light conditions on biomass accumulation and polyphenol production was investigated during a 5-week incubation in ½SH medium supplemented with half the content of vitamins and 3% sucrose. 

**Figure 5:**
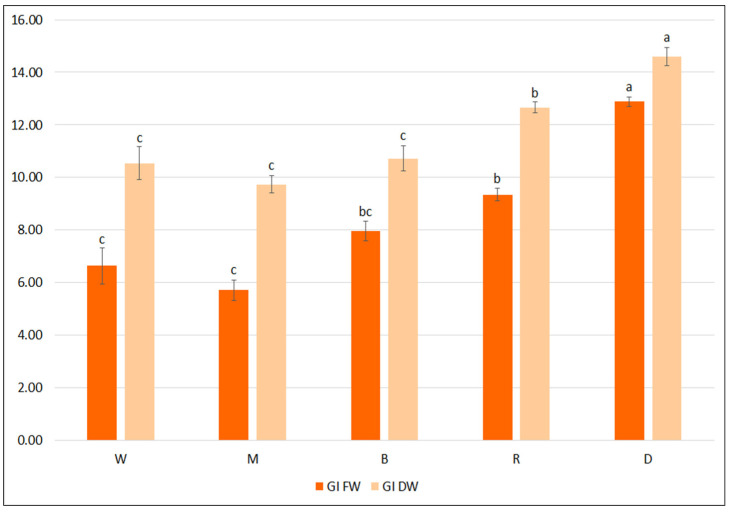
Growth indexes of transformed roots of *S. bulleyana* received for fresh (GI FW) and dry (GI DW) weight during cultivation in ½SH medium with ½ vitamin concentration under different light conditions (W—white, M—mixed, B—blue, R—red, D—dark) for five weeks. The results are mean values ± SE. Different letters indicate statistical differences between samples separately for FW and DW.

All light-grown cultures demonstrated lower FW and DW GIs than those grown in the darkness (Figure 5). Among different LEDs, the highest GI values were achieved under red light: FW 9.3 and DW 12.7. Moreover, the hairy roots grown in the dark demonstrated two-fold higher TPC than in cultures under LEDs. While all hairy roots grown under LEDs demonstrated similar total phenol levels (about 40 mg/g DW), some variation in the production of individual phenolic acids was observed according to light spectra: the highest level of predominant RA was found under blue LEDs (35.2 mg/g DW), and SAK under mixed red/blue LEDs (6.2 mg/g DW) (Table 6). Nevertheless, cultivation under different light conditions does not appear to positively influence biomass accumulation and secondary metabolite production in the hairy roots of *S. bulleyana*.

### 2.5. Growth Kinetics of Hairy Root Culture and Polyphenolic Acid Accumulation

**Figure 6:**
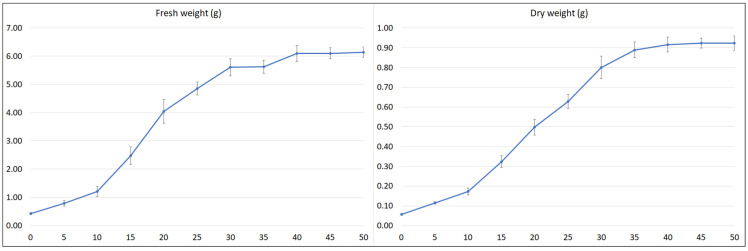
Time course of fresh and dry weight of *S. bulleyana* transformed roots cultivated for 50 days in ½SH liquid medium with ½ vitamin concentration. The results are mean values ± SE.

**Figure 7:**
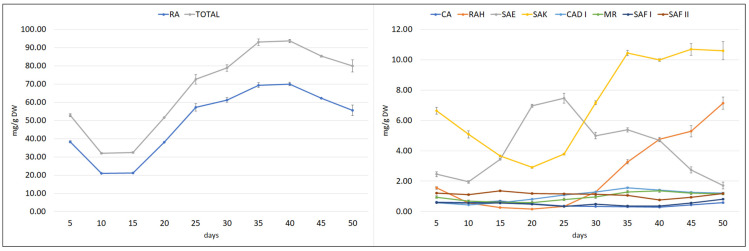
Time course of polyphenolic acid content in *S. bulleyana* transformed roots cultivated for 50 days in ½SH liquid medium with ½ vitamin concentration. CA—caffeic acid, RAH—rosmarinic acid hexoside, SAE—salvianolic acid E, RA—rosmarinic acid, SAK—salvianolic acid K, CAD I—caffeic acid derivative I, MR—methyl rosmarinate, SAF I—salvianolic acid F isomer I, SAF II—salvianolic acid F isomer II. The results are mean values ± SE.

To estimate the optimal collection time of plant material, the growth and polyphenol content of the hairy roots (½SH medium, half vitamin concentration, 3% sucrose, in the dark) was subjected to time-course analysis throughout a 50-day culture cycle (Figure 6 and Figure 7).

After an initial lag period of five days, the culture entered into an exponential growth phase that continued up to day 20. The time taken during this phase for the weight to double (dt) was 6.4 (FW) and 7.1 days (DW), and the specific culture growth rate (µ) was 0.109 (FW) and 0.097 (DW) per day. After 20 days of cultivation, the culture entered a linear growth phase, followed by a stationary phase after another 10 days (FW) or 15 days (DW) (respectively, day 30 and 35 of the growth cycle). The growth of the culture then slowed with maximum biomass at days 45–50. The maximum FW of roots in one cultivation vessel was 6.1 g, and the maximum DW was 0.92 g (Figure 6), i.e., a 14.3-fold increase in FW and 16.1-fold in DW in relation to the inoculum used.

The polyphenol content in the culture also varied in a cycle phase-dependent manner; however, most of the compounds reached the maximum level during the stationary phase. RA content decreased between days 5 and 10, and then its content began to rise sharply from day 15, reaching its highest value of 70 mg/g DW on days 35–40, followed by a gradual decrease (Figure 7). During this period, the MR, CAD I, and SAK values also peaked. SAF I and RAH reached their maximum level in the stationary phase, but later at day 50. The maximum SAE content was observed on day 25, before the stationary phase, while CA peaked on day 15, during the logarithmic phase. The fact that the CA content decreased when the production of other polyphenolic acids intensively increased indicates that it is used as a component for their biosynthesis.

Ultimately, based on the overall productivity of the culture, day 40 was chosen as the optimal day for harvesting the plant material; at this point, 1.07 g of total phenolic acid content, including 0.8 g of RA, was obtained per liter of medium. While greater biomass accumulation was observed later on, the yield of the bioactive metabolite decreased, resulting in lower productivity.

## 3. Discussion

The present work is a follow-up to previous research, in which an efficient methodology for obtaining *S. bulleyana* hairy roots was established. From the obtained clones, the one characterized by the highest biomass and phenol accumulation (clone C4) was taken for a further optimization process. In the present study, we examined for the first time the effects of selected culture conditions, in terms of basal media, and vitamin and sucrose concentrations, as well as light treatment, on improving productivity, with the aim of optimizing polyphenol production in the hairy roots of *S. bulleyana*.

Contemporary in vitro plant cultures used defined media with specified concentrations of individual macro- and microelements and vitamins to obtain reproducible results. The present study used four popular basal media for root cultivation, both with full (MS, WP, B5, SH) and half macro- and microelement contents (½MS, ½WP, ½B5 and ½SH).

The best results were revealed for *S. bulleyana* roots cultivated in SH basal media (SH and ½SH). These media have also been found to be particularly advantageous for the hairy roots of *Levisticum officinale,* *Pimpinella anisum*, and *Angelica gigas* [28,29,30,31]. Both SH and ½SH are characterized by the lowest calcium (Ca^2+^) content among the used media. Calcium is an essential element for plant metabolic processes, although its high level could result in phosphate precipitation, the disruption of related metabolic pathways, and interference with Mg^2+^ function [31]. These media also contain higher levels of micronutrients such as cobalt and iodine in comparison to the others but lower levels of molybdenum and zinc. It is possible that changes in calcium, molybdenum, and zinc ion concentration may have a greater influence on changes in secondary metabolite production in the *S. bulleyana* culture rather than plant growth. For instance, the ½B5 medium demonstrated half the accumulation of root biomass of SH, despite having similar calcium, molybdenum, and zinc contents.

Moreover, the SH media were distinguished by an extremely low ratio of NH_4_^+^ to NO_3_^−^ ions. Nitrogen in SH media is supplied primarily in the form of NO_3_^−^, and NH_4_^+^ ions account only for about 10% of the total amount of nitrogen ions supplied, while that value is 30–40% in the case of the other media used in this study. Some researchers suggest that low total nitrogen content in the medium is beneficial for the growth of hairy roots; such conclusions have been reported for cultures of the medicinal plants *Anisodus acutangulus* [32], *Gmelina arborea* [33] a and *Salvia viridis* [34]. Meanwhile, SH media contain an intermediate content of total nitrogen compared to other media used; thus, the nitrate to ammonium ratio in the medium seems to have a more significant effect on *S. bulleyana* roots than the total amount of nitrogen. George et al. [31] reported that root growth is often promoted by NO_3_^−^ and depressed by NH_4_^+^. Thus, root culture could prefer media containing no NH_4_^+^, or very little. Additionally, the NO_3_^−^/NH_4_^+^ balance significantly influences the pH of the medium: the use of ammonium ions before nitrates acidifies the medium. Consequently, media with higher NH_4_^+^ content could inhibit culture growth to a greater degree. Other researchers also suggest that a high ammonium ion level in the medium can depress the uptake of some metal ions, such as calcium, magnesium, and potassium, which in turn could restrict the nutrient flow into the plant tissue and lower the biomass yield [35]. Some studies, similarly to the present results, also indicated that ammonium ions are necessary for culture growth; however, only a small amount is needed for optimal hairy root growth and further increases in their content resulted in a decrease in the DW of hairy roots [36,37].

Additionally, SH and ½SH media are rich in vitamins; in particular, they contain 5–10 times higher amounts of nicotinic acid compared to the other media used in this experiment. These media are also distinguished by a 10-times higher content of inositol. Despite being a carbohydrate (hexitol), inositol is not regarded as a carbohydrate source for in vitro culture, but rather as a vitamin-like growth enhancer. In particular, inositol promotes cell and protoplast division, may have a role in the uptake, storage, and utilization of ions, as well as possibly participating in stress responses or cell-to-cell communication [38]. Inositol is also important for the storage and transport of auxins, which control the growth of plant tissues. Although it is not itself a hormone, it may be responsible for controlling the functionality of the phytohormones. Inositol has been hypothesized to form conjugates with auxins, thus allowing their safe storage and/or transport. Moreover, these conjugates regulate the availability of active auxins for physiological responses as needed [39].

The vitamin content appears to have an important effect on the biomass and polyphenol accumulation in the hairy roots of *S. bulleyana*. Therefore, the study also estimated the effect of medium vitamin concentration on the culture efficiency. Hence, SH and ½SH media were prepared with half and quarter vitamin concentrations, as well as without vitamins. Despite reducing the vitamin content to half or a quarter, concentrations of nicotinic acid and inositol in SH media were still higher than in the other media used in the experiment, and this change did not adversely affect the growth of the culture. However, in the SH medium, reducing the vitamin content resulted in a gradual decrease in the content of bioactive compounds in hairy roots. In contrast, in the ½SH medium, reducing the vitamin level to half stimulated the production of most polyphenolic acids; only a complete lack of these nutrients drastically reduced the level of secondary metabolites, similar to the SH medium. Thus, ½SH medium with half of the vitamin content demonstrated the greatest accumulation of secondary metabolites in the cultures. In this case, the productivity was 924.4 mg/L for TPC, 741.8 mg/L for dominant RA, and 76.6 mg/L for SAK.

Another parameter tested for the root growth optimization was the sugar content. The choice of carbon source plays an important role in the growth, development, and production of hairy roots and other in vitro cultures [40,41]. Sugar is believed to increase metabolite production in some species due to elevated levels of osmotic stress [42]; however, excessive osmotic pressure could disturb the exchange of components and water between the medium and plant tissues and inhibit their growth. Weremczuk-Jeżyna et al. [41] found 3% to be the optimal sucrose concentration for growth and RA production in *Dracocephalum forrestii* roots. However, other studies found lower (1%) or higher (4%) concentrations to be optimal for the accumulation of biomass and secondary metabolites in transformed roots [40,43]. It can be seen that no specific sucrose concentration is preferable in all cases. Therefore, the present study examined the effects of four different sucrose concentrations (2%, 3%, 4%, 5%).

It was found that media with 2% and 3% sucrose supported the TPC in *S. bulleyana* root culture. Of these, 3% sucrose resulted in a significantly higher accumulation of dry mass, resulting in more efficient productivity of most phenolic acids. However, the levels of SAK and RAH significantly increased in the presence of higher sucrose concentrations (4 and 5%). With regard to RAH, as in the case of other secondary metabolites with a sugar moiety, sucrose enrichment may have increased the glycosidation process. Similar results were also reported for anthocyanin production in *Panax sikkimensis* cultures [44].

LED treatments have also been reported to enhance the biosynthesis of bioactive metabolites in vitro [45,46]. It was observed that light could regulate the expression of genes responsible for the biosynthesis of the phenylpropanoid pathway enzymes that synthesize polyphenolic acids [46,47]. The presence of blue light significantly increased the PAL (phenylalanine ammonia-lyase) activity in strawberry fruits [46], the initial step enzyme of the polyphenol pathway, as well as other enzymes, including shikimate dehydrogenase, tyrosine ammonia-lyase, cinnamate-4-hydroxylase, 4-coumarate/coenzyme A ligase, chalcone synthase, dihydroflavonol-4-reductase, and flavanone-3-β-hydroxylase. Blue light has been found to stimulate the production of polyphenolic acids in some *Lamiaceae* species, such as *Ocimmum basilicum*, *Agastache rugose*, or *Dracocephalum forrestii* [45,48,49].

However, in the present study, all types of LED treatment inhibited secondary metabolite biosynthesis in *S. bulleyana* roots compared to roots cultivated in the dark. This is by no means an unusual finding as root growth naturally takes place in the absence of light. Transformed root cultures are also usually cultivated in the dark, and light conditions have little effect on their growth and metabolite accumulation. Such observations were reported in the case of *Dracocephalum moldavica* [50] and *Salvia officinalis* [51]. Different LED treatments also showed no significant effect on the biomass accumulation and content of salvianolic acid B in hairy roots of *Salvia miltiorrhiza*, while only a slight increase in RA levels was observed for those roots exposed to certain types of mixed light, e.g., blue with red, far-red, and green light [52]. In addition, light can even act as a stress factor, inhibiting the growth of root cultures, as observed for *S. bulleyana* culture. Similarly, the *Rhaponticum carthamoides* and *Pimpinella anisum* hairy roots cultivated under light or photoperiod conditions demonstrated impaired growth in comparison to those kept in darkness [29,53].

Finally, based on our detailed analysis of growth and secondary metabolite production, we can propose an optimal harvest time for the plant material. During the 50-day growth period, the fresh weight of the hairy root culture of *S. bulleyana* increased 14-fold and the dry weight 16-fold, reaching a maximum on days 40–50. The highest total phenol content including RA level was achieved during the stationary phase. A study of *Conodopsis pilosula* hairy roots achieved maximum biomass on day 50 of culture, and, similarly to the present study, the highest secondary metabolite content was reached during the stationary phase [54]. In addition, in *Dracocephalum forrestii* and *S. viridis* culture, the greatest polyphenolic acid accumulation was also recorded in the stationary phase [34,41].

Ultimately, the optimized *S. bulleyana* hairy roots accumulated 93.6 mg TPC/g DW during 40 days of cultivation; this value was more than twice that obtained in the same plant material before optimization (39.6 mg/g DW) [25] and four times higher than in the roots of field-grown plants in the second year of cultivation [4]. Furthermore, the accumulation of RA in the optimized *S. bulleyana* culture on day 40 (70 mg/g DW) was almost twice as high as was found for clone C4 before optimization [25] and eight times higher compared to the roots of the mother plant [4]. This amount of RA was many times higher than noted in the roots of 26 other field-grown species of Chinese sage used as Danshen [2]; it was also higher than observed in the roots of some other *Lamiaceae* species appreciated for their high RA content, such as lemon balm, mint, rosemary, and oregano [55]. Only a few papers have reported similarly high RA content in other optimized transformed root cultures [50,56,57,58].

The presented modifications of the cultivation conditions also allowed for the increased accumulation of other phenolic acids in the transformed roots of *S. bulleyana*. The optimization resulted in a seven-fold increase in the production of SAE, an eighteen-fold increase in RAH, and a two-fold increase in MR in comparison to the original unmodified roots [25], although the maxima for individual metabolites were not reached on the same growth day. On the other hand, SAK was the only compound whose level, even after optimization, was slightly lower than in the roots of the field plant [4]. However, also for this compound, the selection of appropriate cultivation conditions and harvest time increased its content several times. It is also noteworthy that the *S. bulleyana* culture grown in vitro requires a much shorter time to harvest (40 days vs. 2 years), does not degrade the crop during root harvesting, and is free from microbial and other environmental contaminations that are frequently encountered in the field-cultivated plants.

It was also demonstrated during the study, that TOPSIS analysis is a suitable and convenient tool for facilitating the optimization of growth conditions of in vitro plant cultures. By selecting a couple of the most relevant optimization aims, it is possible to indicate an objective solution approaching the ideal scenario as closely as possible. Thus, this approach might be recommended for a wider application in biotechnological studies.

## 4. Materials and Methods

### 4.1. Plant Material

Hairy roots of *S. bulleyana* were obtained as described earlier, using *Rhizobium rhizogenes* (formerly *Agrobacterium rhizogenes*) A4 strain [25]. The roots were cultivated in 80 mL of WP (wood plant) [59] liquid medium on a rotary shaker at 70 rpm at 24 °C, in the dark, and transferred into fresh medium every five weeks. Of the clones investigated by Wojciechowska et al. [25], clone C4 demonstrated the highest growth and polyphenolic compounds production and so was selected for the present study.

### 4.2. Growth Estimation

After the end of the growth cycle, the fresh (FW) and dry (DW) weights of cultures were determined from three flasks from the same treatment. FW (g) was defined immediately after draining off the liquid medium from hairy roots. The material was then frozen and lyophilized and the DW (g) was estimated. The growth index (GI) was expressed as FW(DW) − (FWi(DWi)/FWi(DWi)), where FWi(DWi) was the fresh (dry) weight of the inoculum used to subculture the roots, and FW(DW) the fresh (dry) weight after treatment.

### 4.3. Phytochemical Analysis

The extracts were prepared and analyzed quantitatively according to Wojciechowska et al. [25]. In brief, 100 mg of lyophilized and micronized plant material was extracted once with 30 mL and then twice with 15 mL of methanol: water (4:1 *v*/*v*) using UD-20 ultrasonic disintegrator at 40 °C for 15 min. After blending and evaporation under reduced pressure, extracts were dissolved in 80% methanol and analyzed using an Elite LaChrom Hitachi system supplied with an Ascentis Express C-18 column (7.5 cm × 4.6 mm, 2.7 μm; Supelco, Bellefonte, PA, USA). An aqueous solution of orthophosphoric acid (0.5%) (solvent A, *w*/*w*) and acetonitrile (solvent B) was the mobile phase. The course of the analysis was described earlier [25]. The compound level was expressed as mg/g DW (dry weight) of plant material. Total phenolic content (TPC) was obtained as the sum of contents of all quantified polyphenols.

### 4.4. Effect of Basal Medium on Growth and Polyphenolic Acid Accumulation

Four different standardized basal media were used for hairy root cultivation: WP, SH (Schenk and Hildebrandt) [60], MS (Murashige and Skoog) [61], and B5 (Gamborg et al.) [62] with full- or half-strength macro- and microelement concentrations (½WP, ½SH, ½MS, ½B5). The culture (0.426 g FW, and 0.057 g DW) was transferred into 300 mL Erlenmeyer flasks containing 80 mL medium. Transformed roots were cultivated in the dark, on a rotary shaker at 70 rpm at 24 °C. After five weeks of cultivation, three flasks of each medium were collected to determine the culture biomass accumulation and secondary metabolite production. The experiment was performed three times (passage 32–34).

### 4.5. Effect of Vitamin Content on Growth and Polyphenolic Acid Accumulation

The effect of the vitamin concentration for hairy roots of *S. bulleyana* grown in SH and ½SH medium was investigated. The basal medium was supplemented with full (FV), half (½V), and one-quarter strength (¼V) of vitamins, the standard amount of which for SH and ½SH is: 5.5 mg/L nicotinic acid, 5 mg/L thiamine hydrochloride, 0.5 mg/L pyridoxine hydrochloride, and 1000 mg/L myo-inositol. In the experiment, SH and ½SH basal media were also used without vitamins. Cultures were harvested after five weeks of growth in the same conditions as described earlier. After this time, FW and DW, expressed as GI (growth index), and polyphenol accumulation were estimated. The experiment was performed three times (passages 34–36).

### 4.6. Effect of Sucrose Content on Growth and Polyphenolic Acid Accumulation

To investigate the influence of sugar content on biomass accumulation and bioactive compound production, hairy root culture was cultivated in ½SH liquid medium containing half-strength vitamins, and 2%, 3%, 4%, or 5% sucrose. After five weeks of cultivation in the dark, on a rotary shaker at 70 rpm at 24 °C, three flasks of each treatment were harvested. The FW and DW GI and polyphenol content was examined. The experiment was performed three times (passages 37–39).

### 4.7. Effect of Light Condition on Growth and Polyphenolic Acid Accumulation

Finally, to test the effect of the light environment, the hairy roots were cultivated in ½SH medium with half-strength vitamins and 3% sucrose under different light conditions. The culture was grown in darkness or under a photoperiod (16 h/8 h light/dark) under different LEDs (light emitting diodes): white (390–760 nm), blue (430 nm), red (670 nm), mixed (70% red/30% blue). Hairy roots were maintained on a rotary shaker at 70 rpm at 24 °C for five weeks. Following this, three flasks of each treatment were collected and culture FW and DW were measured to calculate GI, and the polyphenol content was established. The experiment was established three times (passages 40–42).

### 4.8. Growth and Production Studies

For growth kinetics analysis, the roots were transferred into 80 mL of ½SH liquid medium containing half-strength of vitamins and 3% sucrose. The culture was maintained on a rotary shaker at 70 rpm at 24 °C in darkness. The plant material was harvested at five-day intervals over a 50-day culture cycle. The FW and DW were determined, and polyphenolic content was evaluated. Biomass increments were expressed as GI. The procedure was repeated three times (passages 46–48).

### 4.9. TOPSIS Analysis

The TOPSIS analysis was performed according to Ataei [27]. The initial decision matrix (X) was constructed using the selected parameters of culture growth and productivity, i.e., GI for DW, TPC, content of RA, and content of SAK. The X was then normalized ($\bar{x}$) using the following formula

$${\bar{x}}_{ij\;}=\frac{x_{ij}}{\sqrt{\sum_{j}x_{ij}}}$$

where *i* designates the row of the matrix (a variant of medium) and *j* the column of the matrix (particular parameter of culture growth and productivity). A weighted normalized decision matrix (*v*) was then obtained from $\bar{x}$ using the following formula

$$v_{ij}=x_{ij}\times w_{j}$$

where *w_j_* is a relative weight of a particular parameter indicating its importance on a scale from 0 to 1 such that

$$\underset{j}{\sum }w_{j}=1$$

The ideal best (*V^+^*) and ideal worst (*V^−^*) values for each parameter *j* were obtained as follows

$$V_{j}^{+}=\max\left(v_{j}\right)$$

$$V_{j}^{-}=\min\left(v_{j}\right)$$

The Euclidean distance from ideal best and ideal worst for each medium variant *i* were calculated according to formulas

$$S_{i}^{+}=\sqrt{\underset{i}{\sum }{\left(v_{ij}-V_{j}^{+}\right)}^{2}}$$

$$S_{i}^{-}=\sqrt{\underset{i}{\sum }{\left(v_{ij}-V_{j}^{-}\right)}^{2}}$$

Finally, the performance score for each medium variant *i* was calculated using the following formula

$$P_{i}=\frac{S_{i}^{-}}{S_{i}^{+}+S_{i}^{-}}$$

The variants with the highest performance score were treated as optimal.

### 4.10. Data Analysis

All data were expressed as the mean ± standard error (SE) of three experiments. The mean values subjected to ANOVA, followed by Tukey’s *post hoc* test using Statistica 13.1 PL for Windows (StatSoft Inc., Krakow, Poland) with *p* ≤ 0.05 were considered statistically significant. Different letters applied in figures and tables indicate statistical differences between samples, and the letter “a” is always assigned to the highest value for a given parameter. The calculations for TOPSIS analysis were performed using Microsoft Excel 2019 software (Microsoft, Redmond, Washington, DC, USA).

## 5. Conclusions

Our findings revealed that *S. bulleyana* hairy roots demonstrate the greatest growth and provide the highest polyphenolic yield when cultivated in the dark in ½SH liquid medium containing half the vitamin levels and 3% sucrose. The greatest TPC was achieved on day 40 of culture: 93.6 mg/g DW, corresponding to 1072.7 mg/L. The promising levels of rosmarinic acid (801.7 mg/L), salvianolic acid K (114.4 mg/L), and salvianolic acid E (53.8 mg/L) were also noted. The total phenolic acid level was four times higher than that noted in the roots of field-grown plants in the second year of cultivation. Due to its high secondary metabolite level and fast growth, this stable root culture offers the possibility of the year-round production of polyphenolic acids for food, pharmaceutical, and cosmetic applications. Hairy root cultivation reduces our dependence on plants growing in their natural habitat and ensures the high-scale production and year-round availability of unpolluted raw material without being affected by seasonal variations.

## Figures and Tables

**Table 1:** Polyphenolic acid content in transformed roots of *S. bulleyana* cultured in different media after five weeks.

Medium Type	WP	½WP	SH	½SH	MS	½MS	B5	½B5
CA	0.67 ± 0.14 a	0.51 ± 0.01 ab	0.58 ± 0.01 ab	0.59 ± 0.01 a	0.14 ± 0.01 d	0.32 ± 0.01 c	0.50 ± 0.01 ab	0.42 ± 0.08 abc
RAH	0.46 ± 0.03 c	0.27 ± 0.02 d	2.34 ± 0.03 a	1.03 ± 0.01 b	0.09 ± 0.01 e	0.19 ± 0.03 de	0.21 ± 0.04 d	0.21 ± 0.01 d
SAE	1.64 ± 0.25 bc	3.02 ± 0.02 a	2.19 ± 0.02 b	1.43 ± 0.03 c	0.76 ± 0.08 d	0.94 ± 0.22 cd	0.59 ± 0.07 d	1.04 ± 0.23 cd
RA	42.81 ± 2.47 c	41.88 ± 0.07 c	62.17 ± 0.91 a	49.45 ± 0.62 bc	31.04 ± 2.41 d	52.59 ± 0.63 b	55.01 ± 2.52 ab	59.23 ± 1.36 ab
SAK	4.78 ± 0.30 c	3.94 ± 0.02 d	8.08 ± 0.09 a	5.70 ± 0.05 b	0.68 ± 0.13 fg	0.60 ± 0.04 g	1.07 ± 0.02 ef	1.41 ± 0.16 e
CAD I	0.44 ± 0.02 d	0.53 ± 0.01 cd	1.00 ± 0.02 b	1.15 ± 0.01 a	0.58 ± 0.01 c	0.96 ± 0.02 b	0.47 ± 0.02 d	0.55 ± 0.01 c
MR	0.59 ± 0.03 b	0.62 ± 0.01 b	1.14 ± 0.01 a	0.75 ± 0.01 b	0.67 ± 0.09 b	1.03 ± 0.28 ab	0.81 ± 0.08 ab	0.88 ± 0.03 b
SAF I	0.46 ± 0.16 ab	0.96 ± 0.01 a	0.36 ± 0.01 b	0.51 ± 0.02 b	0.32 ± 0.04 b	0.34 ± 0.073 b	0.26 ± 0.08 b	0.22 ± 0.12 b
SAF II	1.43 ± 0.10 b	1.83 ± 0.01 a	1.31 ± 0.03 b	1.55 ± 0.02 b	0.47 ± 0.03 d	0.77 ± 0.06 cd	1.03 ± 0.06 bc	0.74 ± 0.16 cd
TOTAL	53.29 ± 2.60 c	53.54 ± 0.03 c	79.18 ± 1.07 a	62.15 ± 0.73 b	34.76 ± 2.22 d	57.73 ± 0.37 c	59.94 ± 2.45 bc	64.69 ± 1.48 b

CA—caffeic acid, RAH—rosmarinic acid hexoside, SAE—salvianolic acid E, RA—rosmarinic acid, SAK—salvianolic acid K, CAD I—caffeic acid derivative I, MR—methyl rosmarinate, SAF I—salvianolic acid F isomer I, SAF II—salvianolic acid F isomer II. The results are mean values ± SE. Different letters indicate significant differences between samples.

**Table 2:** Polyphenolic acid content in transformed roots of *S. bulleyana* cultured in SH and ½SH medium supplemented with different vitamin concentrations (FV—full vitamin, ½FV—half-strength of vitamins, ¼FV—quarter-strength of vitamins, 0V—without vitamins) after five weeks.

Medium	SH FV	SH ½V	SH ¼V	SH 0V	½SH FV	½SH ½V	½SH ¼V	½SH 0V
CA	0.33 ± 0.01 de	0.31 ± 0.01 e	0.38 ± 0.01 cd	0.51 ± 0.01 b	0.58 ± 0.01 ab	0.35 ± 0.01 de	0.41 ± 0.01 c	0.52 ± 0.03 ab
RAH	0.15 ± 0.01 e	0.14 ± 0.01 e	0.08 ± 0.01 e	0.13 ± 0.02 e	1.02 ± 0.01 b	1.47 ± 0.11 a	0.61 ± 0.01 c	0.33 ± 0.01 d
SAE	3.50 ± 0.04 a	2.92 ± 0.05 bc	2.64 ± 0.05 c	0.82 ± 0.06 e	1.40 ± 0.05 d	3.75 ± 0.14 a	3.14 ± 0.03 b	0.23 ± 0.03 f
RA	66.20 ± 0.36 a	61.68 ± 0.41 b	51.93 ± 0.42 c	25.76 ± 1.24 d	48.79 ± 1.01 c	64.02 ± 0.95 ab	62.35 ± 0.50 b	22.80 ± 0.39 d
SAK	3.01 ± 0.03 e	1.78 ± 0.02 f	1.52 ± 0.03 f	5.94 ± 0.14 bc	5.59 ± 0.13 c	6.61 ± 0.11 b	3.74 ± 0.04 d	7.84 ± 0.36 a
CAD I	0.61 ± 0.01 c	0.49 ± 0.01 d	0.50 ± 0.01 d	0.17 ± 0.01 f	1.14 ± 0.01 a	1.12 ± 0.04 a	0.72 ± 0.02 b	0.24 ± 0.01 e
MR	1.34 ± 0.01 a	0.95 ± 0.01 c	0.82 ± 0.01 e	0.80 ± 0.02 e	0.74 ± 0.01 ab	1.14 ± 0.07 b	0.90 ± 0.01 cd	0.87 ± 0.01 d
SAF I	0.48 ± 0.03 b	0.42 ± 0.03 b	0.67 ± 0.01 a	0.46 ± 0.04 b	0.49 ± 0.02 b	0.27 ± 0.05 d	0.31 ± 0.01 c	0.44 ± 0.01 b
SAF II	1.26 ± 0.01 cd	1.32 ± 0.03 c	1.70 ± 0.04 a	1.08 ± 0.05 de	1.52 ± 0.03 ab	1.05 ± 0.07 e	1.30 ± 0.02 c	1.41 ± 0.06 bc
TOTAL	76.87 ± 0.45 a	70.01 ± 0.52 b	60.24 ± 0.43 c	35.67 ± 1.22 d	61.26 ± 1.25 c	79.78 ± 0.88 a	73.47 ± 0.59 b	34.68 ± 0.33 d

CA—caffeic acid, RAH—rosmarinic acid hexoside, SAE—salvianolic acid E, RA—rosmarinic acid, SAK—salvianolic acid K, CAD I—caffeic acid derivative I, MR—methyl rosmarinate, SAF I—salvianolic acid F isomer I, SAF II—salvianolic acid F isomer II. The results are mean values ± SE. Different letters indicate statistical differences between samples.

**Table 3:** The final weighted normalized decision matrix and calculations of performance score for SH medium with different strength and different vitamin concentrations.

Medium	Weighted Normalized Culture Parameters				TOPSIS Parameters		
	GI DW	TPC	RA	SAK	S^+^	S^−^	P
SH FV	0.1093	0.1067	0.1105	0.0532	0.0854	0.1264	0.5967
SH ½V	0.1014	0.0972	0.1030	0.0314	0.1085	0.1096	0.5026
SH ¼V	0.0986	0.0836	0.0867	0.0269	0.1178	0.0927	0.4404
SH 0V	0.0280	0.0495	0.0430	0.1049	0.1267	0.0783	0.3819
½SH FV	0.0962	0.0850	0.0814	0.0988	0.0571	0.1143	0.6670
½SH ½V	0.1087	0.1108	0.1069	0.1168	0.0220	0.1525	**0.8737**
½SH ¼V	0.0880	0.1020	0.1041	0.0661	0.0763	0.1113	0.5933
½SH 0V	0.0307	0.0481	0.0381	0.1385	0.1239	0.1117	0.4741

SH—full SH medium, ½SH—half-strength SH medium, FV—full vitamin, ½FV—half-strength of vitamins, ¼FV—quarter-strength of vitamins, 0V—without vitamins, GI DW—growth index for dry weight, TPC—total phenolic content, RA—rosmarinic acid, SAK—salvianolic acid K, S^+^—Euclidean distance from ideal best, S^−^—Euclidean distance from ideal worst, P—performance score.

**Table 4:** Polyphenolic acid content in transformed roots of *S. bulleyana* cultured in ½SH medium with ½ vitamin concentration supplemented with different sucrose concentrations (2–5%) after five weeks.

Sucrose Content	2%	3%	4%	5%
CA	0.43 ± 0.01 a	0.27 ± 0.01 c	0.33 ± 0.01 b	0.32 ± 0.01 b
RAH	0.66 ± 0.02 d	3.01 ± 0.04 c	4.63 ± 0.02 a	4.29 ± 0.04 b
SAE	5.00 ± 0.14 a	3.23 ± 0.04 b	2.18 ± 0.02 c	2.14 ± 0.03 c
RA	83.48 ± 1.32 a	72.68 ± 0.99 b	58.91 ± 0.36 c	56.25 ± 0.62 c
SAK	6.99 ± 0.14 d	9.86 ± 0.12 c	11.70 ± 0.09 b	12.53 ± 0.11 a
CAD I	0.75 ± 0.03 a	0.77 ± 0.02 a	0.57 ± 0.01 b	0.52 ± 0.01 b
MR	1.33 ± 0.03 b	1.45 ± 0.02 a	1.14 ± 0.01 c	1.22 ± 0.02 c
SAF I	0.61 ± 0.02 a	0.38 ± 0.02 b	0.34 ± 0.01 b	0.28 ± 0.01 c
SAF II	1.17 ± 0.05 a	0.81 ± 0.02 b	0.89 ± 0.01 b	0.71 ± 0.01 c
TOTAL	100.43 ± 1.23 a	92.45 ± 1.04 b	80.71 ± 0.45 c	78.26 ± 0.80 c

CA—caffeic acid. RAH—rosmarinic acid hexoside. SAE—salvianolic acid E. RA—rosmarinic acid. SAK—salvianolic acid K. CAD I—caffeic acid derivative I. MR—methyl rosmarinate. SAF I—salvianolic acid F isomer I. SAF II—salvianolic acid F isomer II. The results are mean values ± SE. Different letters indicate statistical differences between samples.

**Table 5:** The final weighted normalized decision matrix and calculations of performance score for media with different sucrose concentration.

Sucrose Content	Weighted Normalized Culture Parameters				TOPSIS Parameters		
	GI DW	TPC	RA	SAK	S^+^	S^−^	P
2%	0.0858	0.1420	0.1519	0.0833	0.085	0.0587	0.4085
3%	0.1300	0.1307	0.1322	0.1175	0.0402	0.0665	**0.6232**
4%	0.1372	0.1141	0.1072	0.1394	0.0537	0.0763	0.5869
5%	0.1393	0.1106	0.1023	0.1493	0.0587	0.085	0.5915

**Table 6:** Polyphenolic acid content in transformed roots of *S. bulleyana* cultured in ½SH medium with ½ vitamin concentration and 3% sucrose under different light treatments (white, mixed, blue, red, and dark) after five weeks.

Light	White	Mixed	Blue	Red	Dark
CA	0.63 ± 0.01 b	0.68 ± 0.01 a	0.60 ± 0.01 b	0.45 ± 0.01 c	0.27 ± 0.01 d
RAH	0.29 ± 0.01 c	0.21 ± 0.01 c	0.23 ± 0.02 c	0.59 ± 0.01 b	4.08 ± 0.61 a
SAE	0.35 ± 0.01 c	0.32 ± 0.02 c	0.46 ± 0.02 c	0.70 ± 0.03 b	4.19 ± 0.54 a
RA	29.08 ± 0.03 c	32.16 ± 0.09 bc	35.18 ± 1.45 b	31.17 ± 1.39 bc	70.05 ± 0.95 a
SAK	5.38 ± 0.14 b	6.20 ± 0.27 b	3.18 ± 0.14 c	4.08 ± 0.23 c	9.79 ± 0.20 a
CAD I	0.31 ± 0.01 b	0.40 ± 0.04 b	0.36 ± 0.02 b	0.42 ± 0.02 b	1.19 ± 0.22 a
MR	0.54 ± 0.01 b	0.61 ± 0.05 b	0.52 ± 0.02 b	0.62 ± 0.02 b	1.37 ± 0.02 a
SAF I	0.61 ± 0.01 b	0.67 ± 0.01 b	0.84 ± 0.02 a	0.51 ± 0.02 c	0.36 ± 0.05 d
SAF II	1.62 ± 0.03 a	1.71 ± 0.01 a	1.68 ± 0.05 a	1.32 ± 0.02 b	0.76 ± 0.07 c
TOTAL	38.80 ± 0.09 b	42.96 ± 0.48 b	43.06 ± 1.75 b	39.88 ± 1.70 b	92.06 ± 0.79 a

CA—caffeic acid. RAH—rosmarinic acid hexoside. SAE—salvianolic acid E. RA—rosmarinic acid. SAK—salvianolic acid K. CAD I—caffeic acid derivative I. MR—methyl rosmarinate. SAF I—salvianolic acid F isomer I. SAF II—salvianolic acid F isomer II. The results are mean values ± SE. Different letters indicate statistical differences between samples.

## Data Availability

Data are contained within the article.
